# Identification and Differentiation of the Twenty Six Bluetongue Virus Serotypes by RT–PCR Amplification of the Serotype-Specific Genome Segment 2

**DOI:** 10.1371/journal.pone.0032601

**Published:** 2012-02-28

**Authors:** Narender S. Maan, Sushila Maan, Manjunatha N. Belaganahalli, Eileen N. Ostlund, Donna J. Johnson, Kyriaki Nomikou, Peter P. C. Mertens

**Affiliations:** 1 Arbovirus Molecular Research Group, Vector-Borne Viral Diseases Programme, Institute for Animal Health, Woking, Surrey, United Kingdom; 2 National Veterinary Services Laboratories, Veterinary Services, Animal and Plant Health Inspection Service, U.S. Department of Agriculture, Ames, Iowa, United States of America; Texas Veterinary Medical DIagnostic Laboratory-Amarillo, Texas A&M System, United States of America

## Abstract

Bluetongue (BT) is an arthropod-borne viral disease, which primarily affects ruminants in tropical and temperate regions of the world. Twenty six bluetongue virus (BTV) serotypes have been recognised worldwide, including nine from Europe and fifteen in the United States. Identification of BTV serotype is important for vaccination programmes and for BTV epidemiology studies. Traditional typing methods (virus isolation and serum or virus neutralisation tests (SNT or VNT)) are slow (taking weeks, depend on availability of reference virus-strains or antisera) and can be inconclusive. Nucleotide sequence analyses and phylogenetic comparisons of genome segment 2 (Seg-2) encoding BTV outer-capsid protein VP2 (the primary determinant of virus serotype) were completed for reference strains of BTV-1 to 26, as well as multiple additional isolates from different geographic and temporal origins. The resulting Seg-2 database has been used to develop rapid (within 24 h) and reliable RT–PCR-based typing assays for each BTV type. Multiple primer-pairs (at least three designed for each serotype) were widely tested, providing an initial identification of serotype by amplification of a cDNA product of the expected size. Serotype was confirmed by sequencing of the cDNA amplicons and phylogenetic comparisons to previously characterised reference strains. The results from RT-PCR and sequencing were in perfect agreement with VNT for reference strains of all 26 BTV serotypes, as well as the field isolates tested. The serotype-specific primers showed no cross-amplification with reference strains of the remaining 25 serotypes, or multiple other isolates of the more closely related heterologous BTV types. The primers and RT–PCR assays developed in this study provide a rapid, sensitive and reliable method for the identification and differentiation of the twenty-six BTV serotypes, and will be updated periodically to maintain their relevance to current BTV distribution and epidemiology (http://www.reoviridae.org/dsRNA_virus_proteins/ReoID/rt-pcr-primers.htm).

## Introduction

Bluetongue (BT) is an arthropod-transmitted hemorrhagic disease of wild and domestic ruminants. It is enzootic in many tropical, subtropical and some temperate regions, including much of the Americas, Africa, southern Asia and northern Australia, coincident with the geographic distribution and seasonal activity of competent *Culicoides* vector insects [1], [2], [3]. BT can have major effects on animal health, particularly in naïve ruminant populations, restricting international trade in livestock. It is therefore listed as a ‘notifiable’ disease by the Office International des Epizooties (OIE) [4], [5], [6].

The bluetongue virus (BTV) genome is composed of ten linear segments of double-stranded RNA (dsRNA), which encode eleven distinct virus proteins. Seven of these (VP1 to VP7) are structural components of the icosahedral virus capsid, while four (NS1 to NS4) are non-structural proteins that have been identified in BTV-infected cells [7], [8], [9], [10]. The BTV outer-capsid proteins VP2 and VP5, which are encoded by genome segments 2 and 6 (Seg-2 and Seg-6) respectively, are primarily involved in cell-attachment and entry during the early stages of infection. They also contain epitopes (particularly VP2) that bind neutralising antibodies generated during infection of the mammalian host [11], [12], [13], [14]. The specificity of these reactions in serum neutralisation or virus neutralisation tests (SNT or VNT), shows a close correlation with variations in the amino acid (aa) sequence of VP2, and has been used to identify 26 distinct serotypes (including the recent identification of BTV-25 from Switzerland and BTV-26 from Kuwait) [15], [16], [17], [18], [19], [20].

Phylogenetic analyses of Seg-2 show that it represents the least conserved region of the BTV genome, separating primarily into 26 distinct clades that accurately reflect virus types identified in VNT or SNT (with <32% nucleotype (nt) sequence variation within type: 28.5–59.5% variation between types) [17], [21]. Full genome sequence analyses of multiple BTV isolates from different geographic regions has identified distinct ‘regional groups’ (topotypes): including the major ‘eastern’ and ‘western’ lineages (topotypes) identified by Maan et al [18], [21], [22]. To a lesser extent regional variations are also evident within individual serotypes, with <21.8% nt variation in Seg-2 between strains belonging to the same topotype and serotype. The ‘eastern’ group includes BTV isolates from South East Asia, India, China or Australia, while the ‘western’ group includes viruses primarily from Africa and North or South America [16], [23], [24]. There is also evidence for further distinct topotypes represented by certain viruses from China and Australia, as well as the recently discovered BTV types 25 and 26 [18], [19], [22].

Nucleotide sequence based typing methods (including phylogenetic comparisons of Seg-2 and real-time or conventional RT-PCR assays) are becoming increasingly important for the diagnosis and identification of individual BTV serotypes [18], [21], [22]. Due to the significant level of sequence variation that can exist in Seg-2 between BTV isolates from different geographic origins but belonging to the same serotype (up to 32% nt variation), it is becoming increasingly important to identify reference strains for the major topotypes that exist within each serotype. Where appropriate we have therefore added the letters ‘e’ or ‘w’ to the designations for reference or field strain of different BTV serotypes, to indicate that they belong to either the major eastern or western topotype. Recent isolates of BTV-25 and 26 represent additional distinct western and eastern topotypes, that we can identify as ‘w_2_’and ‘e_2_’ respectively [21], [22].

Prior to 1998, BTV had caused only periodic and relatively short-lived epizootics in southern Europe, involving a single serotype on each occasion [25], [26]. However, exotic BTV strains have arrived in Europe almost every year from 1998 to 2011, including a total nine distinct serotypes and viruses belonging to both eastern (e) and western (w) topotypes (BTV-1e, -1w, -2w, -4w, -6w, -8w, -9e, -11w, -16e and 25w_2_) [27], [28], [29], [30], [31]. Consequently outbreaks (most notably those caused by a strain of BTV-8w) spread to almost the whole of Europe, including several North European countries where BTV had not previously been reported (reviewed by [21], [24], [26], [32]).

BTV has recently also expanded its geographic range in other parts of the world. Multiple additional BTV types were identified in Israel, including: BTV-5w, -8w, -15w (in 2006); BTV-24w (in 2008); BTV-2 (in 2010) and BTV-12 in 2011 (the first time these types have been detected in the Mediterranean region) [33]. BTV-1w, -4w, -8w and -16w were detected in Oman during 2009 [34] and BTV-26 was identified in Kuwait during 2010 [18]. The geographic proximity of these outbreaks to Europe suggests that the BTV strains involved could represent future threats to the region. Since 1999 multiple, previously exotic BTV strains (BTV-1w, -3w, -5w, -6w, -14w, -19w, -22w and -24w) were also detected in the south-eastern United States, most likely as a result of northward spread from the Caribbean Basin [35], [36]. More recently in 2008, two further strains of BTV-9, -12, were identified in Florida and Texas respectively [36]. BTV-7w and -2e were also detected in northern Australia during 2007 and 2008 respectively [37], [38].

The inactivated or live-attenuated BTV vaccines that are currently available are serotype-specific [39]. Rapid and reliable identification of virus serotype can therefore play an important part in the design and implementation of appropriate control measures. Identification of BTV ‘type’ also demonstrates conclusively that the virus belongs to the BTV species, confirming any initial diagnoses by other means [40], and can help to map the origins, movement and spread of individual virus strains.

Serum neutralisation tests can also show a broad heterotypic response to multiple BTV serotypes, particularly if the animal tested has previously been infected or vaccinated sequentially with more than one virus type [41], [42], making ‘typing’ difficult. Serological typing methods require access to standardised reagents, including reference antisera, or reference strains for all 26 BTV serotypes, which can be difficult to produce and could themselves represent a potential infection risk.

Sensitive and specific nucleic-acid based typing assays (by RT-PCR and sequencing) that were previously reported for six European serotypes, including BTV-1, -2, -4, -8, -9 and -16 [16], give clear advantages in terms of speed, sensitivity and specificity over established serological tests. Sequencing of the cDNA amplicons generated in these assays and phylogenetic comparisons to other strains that had previously been analysed also made it possible to identify individual virus lineages in a manner that is not currently possible by serological techniques.

The recent availability of full-length Seg-2 sequence data from multiple isolates of individual types, including both eastern and western strains, has facilitated the selection and evaluation of a complete set of ‘serotype-specific’ primers for all 26 BTV serotypes. The specificity of amplification was confirmed in each case by a lack of cDNA products amplified from the RNA of closely related heterologous serotypes within the same Seg-2 nucleotype. The resulting assays which are described here, provide rapid and reliable BTV serotype and strain identification (in less than 24 hrs), and were used to identify incursions of multiple BTV types into Europe and the USA.

## Materials and Methods

### Virus isolates

A total of 240 strains from the 26 different BTV serotypes, were used in this study, including reference strains and both new and historical field isolates from different geographical locations (Table 1, S1 and S2). These viruses were isolated using embryonated chicken eggs (ECE) (UK Home Office licence number PPL 70/6213) and tissue culture techniques (KC cells - originally provided by colleagues at the USDA lab in Laramie or BHK-21 clone 13 (European Collection of Animal cell Cultures [ECACC – 84100501]), from diagnostic samples including the blood/spleen/lymph nodes of sick or dead animals. These samples were not collected for research purposes, but were taken from naturally infected/dead animals in the field, by qualified veterinarians, as part of normal diagnostic testing procedures in the respective countries. Further details of the isolates are provided on the Orbivirus Reference Collection (ORC) web site at [43].

**Table 1:** Field isolates of BTV typed using Seg-2 based serotype-specific RTPCR assays.

BTV serotype	Origin	ID*
BTV-1	Greece	GRE2001/01
BTV-1	Greece	GRE2001/02
BTV-1	Algeria	ALG2006/01
BTV-1	Morocco	MOR2006/06
BTV-1	Oman	OMN2009/01
BTV-1	USA	USA2004/02
BTV-3	USA	USA1999/06
BTV-4	Oman	OMN2009/09
BTV-5	USA	USA2003/05
BTV-6	USA	USA2006/01
BTV-6	Netherlands	NET2008/04
BTV-6	Netherlands	NET2008/05
BTV-6	Netherlands	NET2008/06
BTV-8	Netherlands	NET2006/01
BTV-8	Netherlands	NET2006/02
BTV-8	Netherlands	NET2006/03
BTV-8	Netherlands	NET2006/04
BTV-8	Netherlands	NET2006/05
BTV-8	Netherlands	NET2006/06
BTV-8	Belgium	BEL2006/01
BTV-8	Oman	OMN2009/03
BTV-8	UK	UKG2007/05
BTV-9	Libya	LIB2008/01
BTV-9	Libya	LIB2008/03
BTV-9	Libya	LIB2008/06
BTV-9	Libya	LIB2008/07
BTV-9	Libya	LIB2008/09
BTV-9	India	IND2005/01
BTV-9	India	IND2005/02
BTV-9	India	IND2005/03
BTV-9	India	IND2005/07
BTV-9	India	IND2005/08
BTV-14	USA	USA2003/03
BTV-15	Israel	ISR2006/11
BTV-16	Oman	OMN2009/02
BTV-19	USA	USA2003/04
BTV-22	USA	USA2002/02
BTV-24	USA	USA2007/01,
BTV-24	Israel	ISR2009/04
BTV-25	Switzerland	SWI2008/01
BTV-26	Kuwait	KUW2010/02
BTV-26	Kuwait	KUW2010/03
BTV-4 and -24	Israel	ISR2008/02
BTV-6 and -8	Germany	GER2008/06
BTV-6 and -8	Germany	GER2008/07
BTV-6 and -8	Netherlands	NET2008/09
BTV-6 and -8	Netherlands	NET2008/11
BTV-9 and -16	Turkey	TUR2000/06
BTV-9 and -16	Turkey	TUR2000/07

* Institute for Animal Health, Pirbright (IAH-P) dsRNA virus reference collection number. More information concerning the origins of these isolates is available at http://www.reoviridae.org/dsRNA_virus_proteins/ReoID/BTV-isolates.htm.

### Extraction of Viral dsRNA

Double stranded (ds) RNA was extracted either from BTV infected cells, using Trizol® (Invitrogen, USA) [44], from tissue culture supernatant, or diagnostic samples, (including blood, spleen, lymph nodes or liver) using the QIAamp Viral RNA Mini Kit from Qiagen (Hilden, Germany), according to manufacturer's instructions. Each isolate was processed at different times to help minimize the risk of sample-to-sample contamination.

### Selection of serotype-specific oligonucleotide primers

Comparisons of full-length Seg-2 sequences from different BTV types [17], [18], [45] has facilitated the identification of unique regions (showing intra-typic conservation and hetero-typic variation) for the design of primers as potential targets in serotype specific RT-PCR assays. Wherever possible at least three primer-pairs were designed for each serotype – If isolates were available from both eastern and western topotypes, one pair was designed for amplification of isolates from both origins (A); one pair each for specific amplification of isolates of an eastern origin (E), and one pair from a western origin (W) respectively. Primers are designated as follows: ‘BTV’ representing the bluetongue virus, followed by serotype, ‘2’ representing Seg-2; a number corresponding to the nucleotide position of the primer in Seg-2 of BTV; and ‘F’ or ‘R’ representing the forward or reverse primer respectively. Each primer-pair was evaluated using reference strains of the other serotypes as well as multiple and diverse isolates (where available) of the homologous serotype, and of the most closely related heterologous serotypes (from the same Seg-2 nucleotype) [17], [24].

The primer-sequences selected for each BTV type were compared (*in silico*) to Seg-2 sequence data for multiple strains of the other 25 types, as well as other orbiviruses closely related to BTV (as listed on ORC web site at [46]) to ensure that they would not cross-react.

### Reverse transcription and amplification of Seg-2

Two RT-PCR protocols were evaluated for amplification of BTV Seg-2. These included a single-tube method using a One-Step RT-PCR kit (Qiagen, Courtaboeuf, France), as previously described by Mertens et al. [16]. The second protocol used the SuperScript™ III one-step RT-PCR system (Invitrogen) with high fidelity platinum® Taq, which is designed for sensitive, high-fidelity end-point detection and analysis of RNA templates extracted from blood or cell culture supernatant, as described by Maan et al. [21]. All primer-pairs were used as described previously [21], with annealing conditions −55°C for 30 seconds. After these reactions 5 microlitres of each cDNA product was analyzed by electrophoresis on a 1% agarose gel, stained with ethidium bromide then visualised under UV light. The identification of each BTV serotype was dependent on detection of an amplified product of the predicted size (Table S1 and S2).

### Sequencing of RT-PCR amplicons

Confirmation of BTV type (as indicated by RT-PCR amplification of a cDNA of the expected size) was obtained by direct sequencing of the amplified products using ‘Big dye cycle sequencing kit’ on an ABI 3730 DNA sequence analyzer, and the same primers for sequencing. Analyses and comparisons of the resulting nt sequence data were carried out using BLAST [47], MEGA 5 [48] and Lasergene software (DNASTAR Inc., Madison, Wis.).

### Evaluation of RT-PCR assays for specificity

The original strain of BTV-25 has not yet been grown in cell culture [49]. The serotype-specificity of primers and RT-PCR assays were therefore evaluated using reference strains of the other 25 BTV types. Most of the reference strains used at IAH were isolated in South Africa and belong to a ‘western’ topotype (w), while serotypes 3w and 4w, 10w, 16e, 25w_2_ and 26e_2_ [21], [22], [50] originated from Cyprus, Portugal, Pakistan, Switzerland and Kuwait respectively. The reference strain of BTV-17w was derived from the USA, while BTV-20e, 21e, 23e were isolated in Australia [51], [52], [53]. The primer-pairs were also tested using dsRNA preparations from the most closely related heterologous BTV serotypes in each case (same nucleotype - Maan et al [17], [21]), as well as multiple isolates of other closely related *Orbivirus* species, including currently available African horse sickness virus (AHSV – reference strains of all 9 serotypes), Epizootic haemorrhagic disease virus (EHDV - reference strains of all 7 serotypes), Equine encephalosis virus (EEV - reference strains of all 7 serotypes), Pata virus (PATAV - CAF1968/01), Andasibe virus (ANDV - MAD1979/01), Tilligerry virus (TILV - AUS1978/03), Mitchell River virus (MRV - AUS1971/01), Wallal Mudjinbarry virus (MUDV - AUS1982/03). In each case the Institute for Animal Health, Pirbright (IAH-P) ‘dsRNA virus reference collection number’ is composed of country code, year, and the number of the isolate in that year from that country. More details about these orbiviruses can be found on ORC web site at [46].

## Results

Testing of previously published RT-PCR assays [54], [55] has shown that primers developed using Seg-2 data from a single isolate, may not work reliably (or may give poor amplification) with other isolates of the same type (data not shown), reflecting significant intra-type sequence variation within Seg-2. Therefore, wherever possible full-length Seg-2 sequence data were compared for multiple isolates of each serotype, to identify relatively more conserved regions prior to the design of type specific primers [18], [21], [45]. However, high levels of intra-type variation (e.g. between topotypes) can make it difficult to detect all isolates of a serotype using a single pair of Seg-2 primers. Under these circumstances, multiple primer-pairs were designed that could be used collectively to amplify all variants within the serotype.

### Selection of serotype specific primers for different BTV ‘types’

We previously reported RT-PCR based typing assays for six European BTV serotypes 1, 2, 4, 8, 9 and 16 [16]. Further introductions of exotic serotypes into Australia, Europe and America, have prompted the design of a complete set of Seg-2 amplification primers for typing all 26 BTV serotypes.

Sequence data generated for Seg-2 of multiple field, reference and vaccine strains (where available - Table S1 and S2) were compared to select ‘foot-print’ sequences for primers that could be used to distinguish and detect each of the 26 BTV serotypes. Details of oligonucleotide primer sequences, specific primer positions, level of specificity and the product sizes expected with different sets of serotype-specific primers for each of the 26 BTV serotypes is given in Table S1 and S2. In some cases (e.g. BTV-7, -21 -25 and -26) the number of available virus strains and/or sequences is limited, which (as already discussed) may impose limits on primer type-specificity, that will require future re-evaluation and re-design.

### Evaluation of RT-PCR assays for type specificity

**Figure 1:**
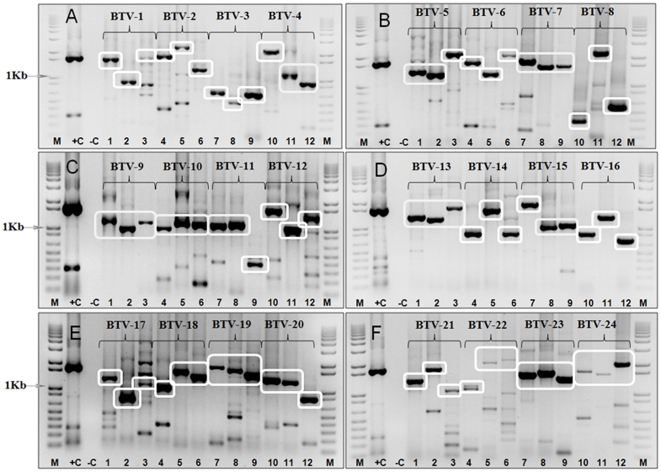
Electrophoretic analysis of cDNA products from Seg-2 of BTV reference strains using ‘type-specific’ primer-pairs for individual serotypes (Panels A–F). Panel A: PCR amplicons were generated from Seg-2 of BTV-1/RSArrrr/01 using primer-pairs ‘1A1’ −1621 bp, ‘1A2’ −864 bp, and ‘1W1’ −1743 bp (lanes 1, 2, and 3 respectively). PCR amplicons were generated from Seg-2 of BTV-2/RSArrrr/02 using primer-pairs ‘2A1’ −1800 bp, ‘2A2’ −2343 bp and ‘2W2’ −1246 bp (lanes 4, 5 and 6 respectively). PCR amplicons were generated from Seg-2 of BTV-3/RSArrrr/03 using primer-pairs ‘3W1’ −648 bp, ‘3W2’ −480 bp and ‘3W3’ −652 bp (lanes 7, 8 and 9 respectively). PCR amplicons were generated from Seg-2 of BTV-4/RSArrrr/04 using primer-pairs ‘4W1’ −2045 bp, ‘4W4’ −1071 bp and ‘4W5’ −929 bp (lanes 10, 11 and 12 respectively). **Panel B:** PCR amplicons were generated using primer-pairs ‘5W1’ −1362 bp, ‘5W2’ −1280 bp and ‘5W3’ −2124 bp from Seg-2 of BTV-5/RSArrrr/05 (lanes 1, 2 and 3 respectively). PCR amplicons were generated from Seg-2 of BTV-6/RSArrrr/06 using primer-pairs ‘6W1’ −1724 bp, ‘6W3’ −1303 bp and ‘6W4’ −2051 bp (lanes 4, 5 and 6 respectively). PCR amplicons were generated from Seg-2 of BTV-7/RSArrrr/07 using primer-pairs ‘7W1’ −1798 bp, ‘7W2’ −1577 bp and ‘7W3’ −1609 bp (lanes 7, 8 and 9 respectively). PCR amplicons were generated from Seg-2 of BTV-8/RSArrrr/08 using primer-pairs ‘8W4’ −363 bp, ‘8W5’ −2216 bp and ‘8W6’ −562 bp (lanes 10, 11 and 12 respectively). **Panel C:** PCR amplicons were generated using primer-pairs ‘9W1’ −1093 bp, ‘9W2’ −961 bp from Seg-2 of BTV-9/RSArrrr/09 (lanes 1 and 2 respectively), and from Seg-2 of BTV-9/BUL1999/01 using primer-pair ‘9E1’ −1105 bp (lane 3). PCR amplicons were generated from Seg-2 of BTV-10/RSArrrr/10 using primer-pairs ‘10W4’ −964 bp, ‘10W5’ −1094 bp and ‘10W6’ −1109 bp (lanes 4, 5 and 6 respectively). PCR amplicons were generated from Seg-2 of BTV-11/RSArrrr/11 using primer-pairs ‘11W4’ −1077 bp, ‘11W5’ −1096 bp and ‘11W6’ −355 bp (lanes 7, 8 and 9 respectively). PCR amplicons were generated from Seg-2 of BTV-12/RSArrrr/12 using primer-pairs ‘12W1’ −1613 bp, ‘12W2’ −892 bp and ‘12W3’ −1326 bp (lanes 10, 11 and 12 respectively). **Panel D:** PCR amplicons were generated from Seg-2 of BTV-13/RSArrrr/13 using primer-pairs ‘13W2’ −1323 bp, ‘13W3’ −1236 bp and ‘13W4’ −1655 bp (lanes 1, 2 and 3 respectively). PCR amplicons were generated from Seg-2 of BTV-14/RSArrrr/14 using primer-pairs ‘14W1’ −850 bp, ‘14W2’ −1581 bp and ‘14W3’ −849 bp (lanes 4, 5 and 6 respectively). PCR amplicons were generated from Seg-2 of BTV-15/RSArrrr/15 using primer-pairs ‘15W1’ −1823 bp, ‘15W2’ −991 bp and ‘15W3’ −1067 bp (lanes 7, 8 and 9 respectively). PCR amplicons were generated from Seg-2 of BTV-16/RSArrrr/16 using primer-pairs ‘16A3’ −851 bp, ‘16E2’ −1288 bp (lanes 10 and 11 respectively) and from Seg-2 of BTV-16/NIG1982/10 using primer-pair ‘16W2’ −726 bp (lane 12). **Panel E:** PCR amplicons were generated from Seg-2 of BTV-17/RSArrrr/17 using primer-pairs ‘17W1’ −1256 bp, ‘17W4’ −689 bp and ‘17W3’ −1090 bp (lanes 1, 2 and 3 respectively). PCR amplicons were generated from Seg-2 of BTV-18/RSArrrr/18 using primer-pairs ‘18W1’ −1021 bp, ‘18W4’ −1556 bp and ‘18W3’ −1381 bp (lanes 4, 5 and 6 respectively). PCR amplicons were generated from Seg-2 of BTV-19/RSArrrr/19 using primer-pairs ‘19W1’ −1787 bp, ‘19W2’ −1680 bp and ‘19W3’ −1494 bp (lanes 7, 8 and 9 respectively). PCR amplicons were generated from Seg-2 of BTV-20/RSArrrr/20 using primer-pairs ‘20E1’ −1324 bp, ‘20E2’ −1257 bp and ‘20E3’ −827 bp (lanes 10, 11 and 12 respectively).**Panel F:** PCR amplicons were generated from Seg-2 of BTV-21/RSArrrr/21 using primer-pairs, and ‘21E3’ −1524 bp, ‘21E2’ −1726 bp and ‘21E1’ −1320 bp (lanes 1, 2 and 3 respectively). PCR amplicons were generated from Seg-2 of BTV-22/RSArrrr/22 using primer-pairs ‘22W1’ −1074 bp, ‘22W2’ −2034 bp and ‘22W3’ −2211 bp (lanes 4, 5 and 6 respectively). PCR amplicons were generated from Seg-2 of BTV-23/RSArrrr/23 using primer-pairs ’23E1’ −1548 bp, ‘23E2’ −1623 bp and ‘23E3’ −1421 bp (lanes 7, 8 and 9 respectively). PCR amplicons were generated from Seg-2 of BTV-24/RSArrrr/24 using primer-pairs ‘24W1’ −1776 bp, ‘24W2’ −1557 bp and ‘24W3’ −2021 bp (lanes 10, 11 and 12 respectively).Lane M: 1 Kb marker. +C is a positive control using RNA from BTV-6/RSArrrr/06, with primer-pair BTV-6/2/301F & BTV-6/2/790R −1631 bp – [21]. −C is a negative water control. For primer position and sequence see Table S1 and S2. The use of type specific primers for BTV-26 was recently published by [18], [22].

Reaction conditions were optimized for each primer pair, as described in the materials and methods section. Each of the 25 BTV reference strains (BTV-1 to 24 and BTV-26) listed in Table S1 and S2, was tested to confirm the specificity of at least three different primer sets for each of 26 BTV serotypes. In most cases no amplified products were detected from heterologous serotypes, even within the same nucleotype, providing an initial validation for the use of these primers to discriminate between BTV types. Each of the primer-pairs generated an abundant cDNA amplicon of the expected size, from RNA of the homologous BTV reference strain, although some also generated additional minor bands of an ‘incorrect’ size in these reactions (Table S1 and S2, Figure 1).

The Seg-2 primers for each BTV serotype were also evaluated for type specificity, by *in silico* comparisons to Seg-2 sequence data for multiple other BTV isolates and other related orbiviruses, as listed on ORC web site at [46] (data not shown). These comparisons indicated no high-similarity binding sites in heterologous BTV serotypes or virus-species that are likely to result in mispriming under the reaction conditions used. The majority of primers listed in Table S1 are therefore thought to be genuinely serotype-specific (as indicated), although novel strains may appear in the future that are more ‘cross-reactive’.

However, some primer-pairs, for the serotypes in nucleotype A (BTV-4, -10, -11, -17, -20 and -24), nucleotype D (BTV-8, -18 and -23), BTV-16 and BTV-26 (as listed in Table S2) were not entirely type-specific. In these cases they also amplified Seg-2 sequences from some of the most closely related heterologous serotypes, generating amplicons of the expected size. However, unlike real-time PCR assays, both the ‘conventional’ type-specific primers and these ‘non-type-specific’ primers can be used to sequence the Seg-2 cDNA amplicons that they generate, providing definitive serotype and strain identification, via phylogenetic comparisons.

### Use of RT-PCR assays for typing field isolates from recent BTV outbreaks

The type-specific RT-PCR assays and sequencing methods described here were used for primary identification or confirmation of serotype, for multiple field strains of BTV. Confirmation of the initial RT-PCR typing results, was obtained by direct sequencing of the amplified products from Seg-2 using the same RT-PCR primers, as sequencing primers. The resultant sequences were subjected to either BLAST analysis or phylogenetic comparisons to Seg-2 sequence data for multiple isolates of the 26 BTV serotypes (including a total of over 300 Seg-2 sequences, including both eastern and western strains). The BTV serotype was confirmed by detection of high levels of sequence identity to reference strains of the relevant type.

The strains identified using these methods included BTV-1 isolates from the Mediterranean region (Table S1 and S2). Two Greek strains were identified as BTV-1e, related to viruses from India (Table 1) [45]. In contrast two incursions in Algeria and Morocco were identified as BTV-1w, one of which spread to south-west France during November 2007 (Table 1) [56].

Type specific RT-PCR assays were also used during August-September 2006 for the primary identification of BTV-8 in clinical samples from animals in the Maastricht region of northern Europe. In August 2007, BTV-8 was again identified in the UK (the first recorded BTV incursion in the UK). Late in 2008/early 2009, BTV-6 was identified in the Netherlands [21], BTV-11 in Belgium [57], BTV-25 in Switzerland [19] and BTV-26 in Kuwait (Table 1) [18].

**Figure 2:**
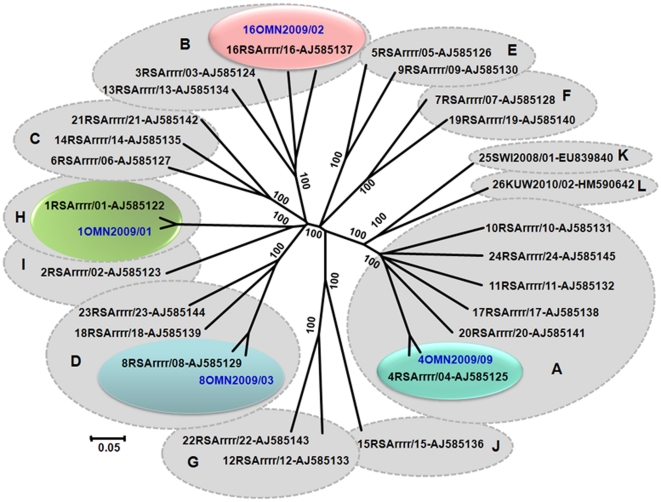
Neighbour-joining tree, showing relationships in Seg-2 between reference and field strains of BTV-1w, 4w, 8w and 16w from Oman. The tree was constructed using distance matrices, generated using the p-distance determination algorithm in MEGA5 (500 bootstrap replicates) [48]. BTV split into 26 distinct groups based on Seg-2 sequences, reflecting serological relationships between virus strains [18]. Reference strains of BTV are shown in black and field isolates from Oman are shown in blue font. Isolate designations: IAH ‘dsRNA virus reference collection number’ composed of country code, year, and the number of the isolate in that year from that country [66]. Full length Seg-2 sequences of BTV-1/OMN2009/01, BTV-4/OMN2009/09, BTV-8/OMN2009/03 and 16/OMN2009/02 showed 95%, 94%, 93% and 73.6% nucleotide (nt) identity to the reference strains of respective serotype, confirming their types and topotypic groups.Scale represents number of substitutions per site. Values at major branching points represent NJ bootstraps.

The assays and primers described here were also used at IAH to identify several BTV isolates sent from America (in 2006–2007), as BTV-1, -3, -5, -6, -14, -19, -22 and -24 respectively [36], types that were previously exotic to the USA. Isolates from India (BTV-9e), Oman (BTV-1w, -4w, -8w and -16w) (Figure 2); Israel (BTV-15w and BTV-24w - [33]) were also identified using these techniques (Table 1). The amplification of BTV-9 from Libya (Table 1 and Table S1) is shown in Figure S1. Data for the amplification and sequencing of many other samples, to identify or confirm serotype, are not shown in this paper.

Unlike previous serological methods, the type specific RT-PCR assays can and have provided direct positive identification of individual serotypes, as part of mixed serotype infections/isolates, including BTV-4w and -24w from Israel; BTV-6w and -8w from Germany and the Netherlands; BTV-9 and -16 from Turkey.

## Discussion

We describe a complete set of primer-pairs, together with an initial evaluation of their use in RT-PCR amplification assays to identify the 26 serotypes of BTV. The virus strains used in this study originated from all over the world, including reference strains, new and historic isolates from Europe, America, the Middle East, Africa, Australia and the Indian subcontinent, helping to demonstrate the relevance of these assays for detection and typing of different BTV topotypes.

Genome segment 2 was chosen as the target for development of serotype specific RT-PCR based assays, because it encodes the main type-specific antigen and outer-capsid protein VP2 of BTV [17], [58], [59], [60]. However, primers previously designed for Seg-2 of American [55] and Australian [54] isolates, in most cases amplified only small or partial products from the RNA of homologous BTV reference strains (same serotype). This reflects the high level of nt sequence variation (up to 32%) that can exist in Seg-2 between different isolates of the same serotype from different geographic regions (different topotypes) [24], [45].

Primers were recently also described for identification of contaminating serotypes in vaccine studies [61]. However, the specificity of amplification and detection was not widely validated with different topotypes, or isolates of the 26 BTV serotypes, leaving some uncertainty concerning their wider specificity.

The specificity, reproducibility and reliability of the RT-PCR assays described here were tested using reference strains of BTV-1 to BTV-26, field strains of the homologous type (representing different topotypes), as well as more distantly related viruses belonging to different *Orbivirus* species (negative controls), as listed on the ORC web site at [46]. For simplicity and ease of use, each of the conventional RT-PCR assays uses the same amplification conditions to generate cDNA amplicons from Seg-2 of distinct virus-lineages and topotypes within each BTV serotype. All of the primer-pairs generated a major cDNA amplicon of the expected size, from RNA of the homologous BTV reference strain. However, in some cases additional cDNA bands were also generated that are not of the size predicted for the amplified BTV Seg-2 sequences (e.g. primer set ‘17W3’ for BTV-17 – Figure 1 (panel E) lane 3; Table S1 and S2). The synthesis of these bands could be reduced by further optimisation for individual primer pairs. However, the initial indication of virus type can also be confirmed by sequence analysis of the cDNA amplicons (using the same primers) and phylogenetic comparisons to previously characterised strains of BTV-1 to BTV-26.

The RT-PCR assays can be applied directly to RNA extracted from diagnostic samples of viraemic blood or tissues, and do not therefore depend on virus isolation, adaptation to growth in cell culture, or neutralisation assays, which can collectively take several weeks to give a reliable identification of virus- type. It can also be difficult to identify different BTV types within a single ‘mixed’ isolate, or identify the specificity of neutralising antibodies from animals infected or vaccinated with more than one serotype, using serological methods.

Conventional and real-time RT-PCR assays have been used routinely since 2006, for the identification of different BTV serotypes in diagnostic samples of tissue, blood, or cell-culture materials, within the BTV reference laboratory at IAH Pirbright [21], [45], [62], [63]. Samples with CT values of <30 by real-time RT-PCR (equivalent to 200 to 1000 copies per ml of blood) also reliably generate cDNA amplicons by these conventional RT-PCR assays targeting Seg-2. Data concerning real-time RT-PCR sensitivity for detection of different topotypes of the BTV serotypes found in Europe [BTV-1, 2, 4, 6, 8, 9, 11 and 16], was supplied by Laboratoire Service International (LSI).

It is difficult to give a direct comparison of either conventional or real-time RT-PCR assays, in terms of sensitivity, to virus isolation or titration in cell culture. The relative infectivity of different BTV strains depends on their level of adaptation to the cell-culture system used. Indeed, many wild type BTV strains (from diagnostic samples) will not grow directly in mammalian cell cultures (e.g. BHK-21 cells). However, in our hands BTV isolation in KC cells (derived from *Culicoides sonorensis*) is usually successful for samples with CT values <30 to 32. The sensitivity of the conventional RT-PCR assays described here is therefore directly comparable to that of virus isolation in KC cells, although it is less affected by sample degradation and bacterial contamination.

Eastern and western strains of the same serotype were available for BTV-1, 2, 9 and 16. ‘Type’ specific ‘A’ (all) primers were identified that would amplify Seg-2 from both eastern and western strains of BTV-1 and 2. However, the different topotypes of BTV-9 and BTV-16 are too divergent for ‘common’ type-specific primers and additional primer pairs were therefore designed to allow amplification of the more diverse strains (E and W) within these types (Table S1 and S2).

RT-PCR assays were tested using RNA derived from infected cell culture material, as well as clinical samples including blood, spleen, lymph nodes and liver. In each case they were reliable and effective for BTV typing. Although these RT-PCR assays are highly sensitive, they detect different virus-specific molecules from those detected by ELISA or competition ELISA (proteins/antigens or antibodies respectively) and they will therefore have different diagnostic applications.

The RT-PCR assays and sequencing primers targeting Seg-2 have already been used to identify multiple isolates from around the world (Table S1 and S2) confirming their sensitivity, specificity, speed and reliability for BTV typing. It has previously been established using serological methods that multiple serotypes of BTV are often present and can co-circulate in the same endemic regions (as seen recently in Europe) [41], [42]. The RT-PCR assays described here have been used to identify several isolates containing mixed BTV types, which would previously have remained untyped by VNT. These observations suggest that co-infection by different types may be a more common features of BTV epidemiology than previously recognised.

The sequences of primers for the amplification and sequencing of Seg-2 from different BTV serotypes are available via the internet at [64]. Some of the primer sets were designed based on only a limited number of isolates (e.g. BTV-7, -18, -21, -25 and -26) and consequently do not include different topotypes. If new isolates of these types are identified that are not detected by the primers listed here (which is considered likely), further sequencing of Seg-2 will support the design and updating of typing primers.

There remains a need to generate sequence data for Seg-2 of BTV vaccine strains, to allow the design of RT-PCR primers to distinguish them as distinct lineages of field strains, as already done for several European serotypes [16]. This would help to identify field transmission of ‘live’ vaccines, as reported for BTV-6 and -11 in northern Europe and BTV-2 in the Mediterranean region [21], [57], [65].

Phylogenetic comparisons of BTV Seg-2 sequences generated using the conventional primers described here will help to determine the worldwide prevalence, distribution and variability of different BTV types and topotypes, improving our understanding of their epidemiology and transmission. The data generated will also help to inform the design and implementation of future control strategies, including vaccination and the development of novel vaccines.

## Supporting Information

Figure S1 **Electrophoretic analysis of cDNA products from Seg-2 of BTV-9 isolates from Libya 2008 using ‘type-specific’ primer-pairs.** PCR amplicons were generated from Seg-2 of LIB2008/01, LIB2008/09, LIB2008/03, LIB2008/07 and LIB2008/06 using primer-pair ‘9W1’ −1093 bp (lanes 4, 6–9 respectively). RNA of BTV-5/RSArrrr/05 was used as a heterologous control (lane 5). Lanes 1 and 2 are positive controls using RNA from BTV-9/RSArrrr/09 and BTV-9RSAvvv1/09 respectively. Lane 3 has non-template negative control.
(TIF)

Table S1 Primer-pairs for type-specific amplification and sequencing of Seg-2 from BTV-1 to BTV-26 in RT-PCR assays.
(XLS)

Table S2 Additional primer-pairs for amplification and sequencing of Seg-2 from BTV-1 to BTV-26 in RT-PCR assays.
(XLS)
