# Supplementary figures and images for: Identification and Differentiation of the Twenty Six Bluetongue Virus Serotypes by RT–PCR Amplification of the Serotype-Specific Genome Segment 2
Source: PLoS One. 2012 Feb 28;7(2):e32601. doi: 10.1371/journal.pone.0032601 (PMC3289656; doi:10.1371/journal.pone.0032601)

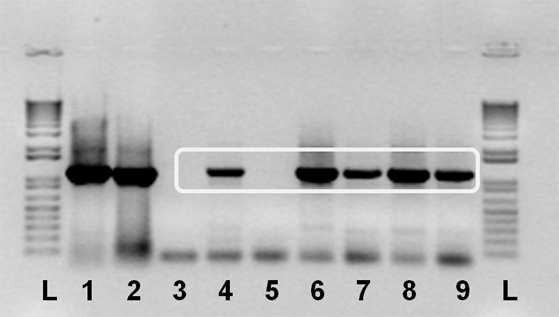

Supplement: Figure S1 — Electrophoretic analysis of cDNA products from Seg-2 of BTV-9 isolates from Libya 2008 using ‘type-specific’ primer-pairs. PCR amplicons were generated from Seg-2 of LIB2008/01, LIB2008/09, LIB2008/03, LIB2008/07 and LIB2008/06 using primer-pair ‘9W1’ −1093 bp (lanes 4, 6–9 respectively). RNA of BTV-5/RSArrrr/05 was used as a heterologous control (lane 5). Lanes 1 and 2 are positive controls using RNA from BTV-9/RSArrrr/09 and BTV-9RSAvvv1/09 respectively. Lane 3 has non-template negative control. (TIF) [file pone.0032601.s001.tif]
